# Human Hibernation

**Published:** 1914-05-23

**Authors:** 


*To the Editor of* The Hospital.

### Human Hibernation.

*To the Editor of* The Hospital.

Sir,—With reference to the letter of "E. L." in your issue of 16th inst., may I say that of all the existing organisations none, from a humane point of view, is more important than that for the prevention of burying people alive? To those who have not studied the facts, and to others who are sceptical as to the existence of trance, catalepsy, or other death-simulacra, sometimes known as human hibernation, such a movement appears to be unnecessary, Utopian, and a waste of energy.

On the other hand, those who carefully examine the evidence brought together by numerous authorities, including professors of medicine, hospital surgeons, and scientific observers, lose their scepticism in the amount of cumulative evidence which besets the path of their researches. Narrow escapes from interment alive, it may be truly said, are almost of everyday occurrence all the world over (especially in countries like France, Spain, Portugal, Italy, Russia, and Turkey, where apparent death is more frequently followed by prompt burial than in these islands), the discoveries often being made during the preparations for the funeral. It may be reasonably inferred from these cases that some, if not many, do not escape, and are buried whilst in a state of suspended animation.

Every session the Association for the Prevention of Premature Burial presents a Bill to Parliament to prevent these terrible tragedies by reforming the present very haphazard, unscientific, and dangerous system of death certification.—I am, Sir, yours respectfully,

May 19, 1914. Burial Reformer.

[Note.—*The letter from Dr. Wilde was published last week through a misunderstanding, which we much regret.*]

